# An effective platform for cancer immunotherapy: pooled knockin targeting for genome engineering

**DOI:** 10.1038/s41392-020-0208-9

**Published:** 2020-06-12

**Authors:** Ke Jin, Long Zhang, Fangfang Zhou

**Affiliations:** 1 Laboratory of Human Diseases and Immunotherapies, West China Hospital, Sichuan University, Chengdu, 610041 China; 2 MOE Laboratory of Biosystems Homeostasis and Protection and Innovation Center for Cell Signaling Network, Life Sciences Institute, Zhejiang University, Hangzhou, 310058 China; 3 Institutes of Biology and Medical Science, Soochow University, Suzhou, 215123 China

**Keywords:** Cancer, Immunotherapy

Recently, a paper published in *Cell* by Theodore L. Roth et al. reported the development of a platform to assess the functional effects of pooled knockin constructs targeting a specific locus in the genome. It also provided a strategy that allows discovery of novel synthetic constructs in the pools to enable engineering T cells with gain of function and promote antitumor activity of T cells in vivo.^1^

**Fig. 1:**
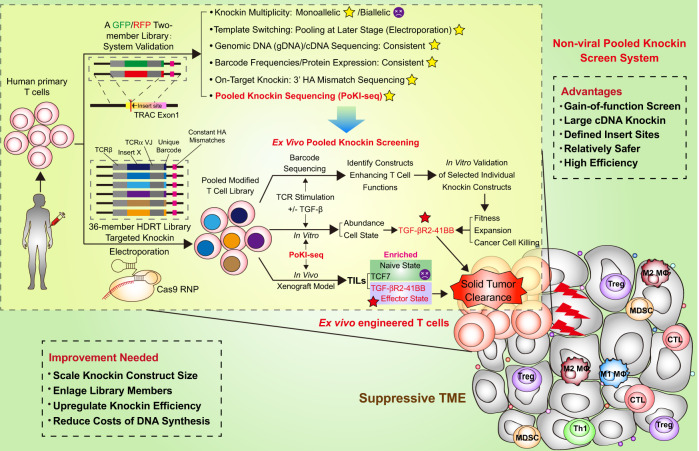
Schematic illustration of the Non-viral Pooled Knockin Screen System. This platform aims to engineer primary human T cells with gain-of-function effects and is promising to accelerate discovery of synthetic constructs with potent antitumor activity in T cells

The immune related components in the tumor microenvironment (TME) are complex. Among them, tumor-infiltrating lymphocytes (TILs) are recognized as one of the key pillars of cancer immunotherapy. Infiltrating T cells, including CD4^+^ and CD8^+^ subsets, participate in coordination in shaping tumor immunity and influencing the fate of cancer. Therefore, developing therapeutic approaches targeting T cells that ultimately enhance the antitumor activity of TILs appears to be particularly important. Immune checkpoint therapy (ICT) performed by blocking CTLA-4, PD-1, or PD-L1 has been demonstrated as an effective strategy for clinical cancer treatment. However, the main obstacle for limiting the effectiveness of ICT in the clinical setting is T cell exhaustion. Tumor cells dictate immunosuppressive components (immune cells such as Treg cells, M2 macrophages, and myeloid-derived suppressor cells (MDSCs) as well as cytokines including TGF-β and IL-10) to orchestrate an immunosuppressive TME, which restrains entry and activity of the effector T cells and hinders ICT (Fig. 1). Particularly, blocking TGF-β in the osseous TME restores antitumor activity of ICT in a bone prostate cancer model.^2^ In addition, cellular therapeutics utilizing CD8^+^ T cells with chimeric antigen receptors (CARs) have also exhibited clinical success against hematopoietic malignancies. Nevertheless, CAR-T therapy still has some disadvantages. First, searching for unique neoantigens in tumors is difficult and time consuming because of tumor heterogeneity accompanied with immunoediting during cancer evolution.^3^ Second, CAR-T has not proven effective in the treatment of solid tumors. Third, the manufacturing process for CAR-T is complex and expensive. Hence, developing new methods for constructing genome-engineered human T cells holds great potential for the next generation of cell-based immunotherapies for cancer treatment.

In 2015, Alexander Marsona group in collaboration with Jennifer Doudna developed a robust CRISPR/Cas9-based technology that enabled both “knockout” and “knockin” genome editing in primary human T cells.^4^ The core elements for this system are Cas9 ribonucleoproteins (RNP), a complex of recombinant Cas9 proteins and an in vitro transcribed sgRNA. For “knockin” editing, a homology-directed repair template (HDRT) is needed. There are several characteristics for this system (Fig. 1). First, this CRISPR/Cas9 genome-targeting technology utilizes electroporation instead of recombinant viral vectors, which allows rapid and efficient insertion of DNA sequences ranging from 2 kb to 3 kb without notable cell toxicity.^1,5^ Moreover, this non-viral genome-targeting method allows the correction of point mutations in the original genome.^4^ Second, during the “knockin” procedure, DNA cassettes can be introduced to specific genomic sites remolding the function of T cells. In the current study, it is by using this system that Alexander Marsona group succeeds in integrating a pool of functional DNA cassettes into the first exon of the T cell receptor (TCR)-α constant region (TRAC). Thereafter, the endogenous TCR locus is replaced with a new TCR that redirects the T cells to recognize a specific cancer antigen. Third, multiple pooled knockin templates with specific barcodes are conducive to the subsequent detection of the abundance of each on-target insert. Combined with the above genome-editing platform, Alexander Marsona group further developed a pooled knockin sequencing (PoKI-seq) system consisting of pooled knockin screening and single cell RNA sequencing (scRNA-seq) to measure cell abundance and cell state ex vivo and in vivo.

It is worth noting that, on-target knockin constructs should be captured before the subsequent experiments because nonhomologous end joining (NHEJ) repair, un-edited target genomic sequences, non-integrated HDRTs, and off-target integrations also exist in engineered T cells. Based on the theories that the majority of HDRT’s homology arm sequences are not incorporated with on-target HDR integrations, and a short-mismatched DNA insert introduced into one homology arm of HDRT would not largely reduce the knockin efficiency, Theodore L. Roth et al. inserted the constant HA mismatches into the 3’ homology arm while keeping the 5’ homology arm of each HDRT identical to the insertion site. By designing an insert-specific forward primer and a genomic site-specific reverse primer, the on-target knockin templates can be captured and identified, while off-target knockin templates can be excluded. Additionally, multiple integrations and template switching should also be avoided during pooled knockin experiments. Hence, they constructed a two-member barcoded library encoding GFP and RFP to validate the system and assess the parameters above (Fig. 1). As a result, they discovered that only a quarter of the engineered cells exhibited multiple integrations. Furthermore, they found that later pooling stages could decrease the frequency of template switching, that is, pooling all subsequent knockin construct libraries at the electroporation stage would significantly reduce the rate of template switching. Ultimately, they found that 9 out of 10 engineered cells were on-target with the correct barcode.

While taking the above parameters into account, Theodore L. Roth et al. enlarged the library to 36 members. These therapeutic knockin constructs in the library were previously described to impact fitness advantages in T cells such as immune checkpoint molecules, suppressive cytokines and apoptotic receptors and so on. First, after a series of ex vivo pooled knockin screenings and sequencing of engineered T cells with TCR stimulation in the presence or absence of TGF-β1, a subset of T cells incorporated with a novel chimeric TGF-βR2-41BB construct showed distinct phenotypes and functions characterized by powerful tumor killing capacity in vitro (Fig. 1). Next, by scRNA-seq using hallmark genes for defining T cell subpopulations in TME, they detected typical phenotypes of TILs, such as naive-like (*CCR7*), proliferation (*MKI67*) and effector (*IL2* and *IFNG*). By abundance analysis, T cells with TGF-βR2-41BB receptor were discovered to be the most enriched effector T cells in TILs. Finally, by adoptive cell transfer of primary human T cells with TGF-βR2-41BB cassette, they demonstrated these cells had vigorous tumor clearance potential in a xenograft melanoma NSG mouse model.

Altogether, satisfactory therapeutic outcomes from cancer immunotherapies cannot be achieved without overcoming the dominant immunosuppressive TME. The targeted non-viral pooled knockin screen system has notable advantages and can be used to discover the effects of gene gain-of-function in T cells, which would potentially enhance their tumoricidal efficacy. Utilizing this extensible pooled knockin platform, we can selectively screen and enrich adaptive T cell subsets in TILs. It also opens new avenues for understanding the functional rules of engineering the human genome, reprogramming the fitness and states of T cells, and providing genetic and cellular cues to enhance cell-based immunotherapies for treating solid tumors.
